# The Effects of Fenobucarb on the Physiology, Behavior, and Growth of Silver Barb (*Barbonymus gonionotus*)

**DOI:** 10.3390/toxics13010012

**Published:** 2024-12-25

**Authors:** Tam Thanh Nguyen, Håkan Berg, Loi Ngoc Nguyen, Yen Thi Hai Nguyen, Cong Van Nguyen

**Affiliations:** 1 Faculty of Fisheries, Nong Lam University, Ho Chi Minh City 700000, Vietnam; nthanhtam@hcmuaf.edu.vn (T.T.N.); nguyenngocloi195@gmail.com (L.N.N.); 2 Department of Physical Geography, Stockholm University, SE-106 91 Stockholm, Sweden; 3 College of Environment and Natural Resources, Can Tho University, 3/2 Street, Can Tho City 900000, Vietnam; nthyen@ctu.edu.vn (Y.T.H.N.); nvcong@ctu.edu.vn (C.V.N.)

**Keywords:** Mekong Delta, acetylcholinesterase, fish toxicology, sublethal effects, rice farming

## Abstract

This study assessed the effects of fenobucarb (F) (1%, 10%, and 20% of the LC_50_-96h value) on the brain cholinesterase (AChE) activity, food intake (FI), feed conversion rate (FCR), and growth of silver barb (*Barbonymus gonionotus*, Bleeker, 1849). It also assessed the AChE inhibition levels that cause the abnormal swimming, behavior, and mortality of silver barb and how the feeding regime affects the recovery rate of the AChE activity. The results showed that the brain AChE inhibition increased with the F concentrations. It peaked after nine hours, at 73.6% and 79.7% for the two highest concentrations, and then the AChE activity started to recover. After 96 h, the inhibition level was still 11.8% in the fish exposed to the two lowest concentrations and 30.5% in the fish exposed to the highest concentrations. Even when placed in clean water, the inhibition level in the fish that were exposed to the highest concentrations and only fed every third day was 32% after 14 days. Although there were no differences in the feed intake at any time, the fish exposed to F had a higher FCR and a lower specific growth rate and weight compared to the control fish at the later stages of the experiment. Thus, although the use of F in rice farming in the Mekong Delta may not lead to direct fish kills, it impacts the growth and health of the fish, which could have negative implications for wild fish populations and the long-term production of healthy fish in the Mekong Delta.

## 1. Introduction

Covering an area of approximately 40,000 km^2^, the Mekong Delta comprises around 12.2% of Vietnam’s total landmass and produces more than half of the country’s rice supply [1,2,3,4]. To achieve the anticipated rise in rice production, numerous farmers now grow three rice crops annually, with significant inputs of fertilizers and pesticides [5]. The high use of pesticides has led to an elevated risk of negative impacts on both environmental and human health [3,6]. The Ministry of Agriculture and Rural Development (MARD) [7] reported that 28,520 tons of commercial pesticides were utilized in the Mekong Delta in 2020, representing 54.9% of the total pesticide usage across the country. On average, 6.3 kg of pesticides are applied per hectare of farmland annually in the Mekong Delta, a figure that is 64.6% higher than the national average [8]. According to MARD [7], the list of pesticides allowed for use in Vietnam includes 1648 active ingredients and 4069 commercial products. One of these is the active ingredient fenobucarb (F), a carbamate pesticide widely used in the Mekong Delta for the control a range of insects, including delphacids, thrips, leafhoppers, and leaf rollers, in rice and other crops. In 2020, F was found in more than 39 pesticides, such as Excel Basa 50EC, Triray 50EC, Vibasa 50EC, and Vitagro 50EC, which are commonly used in the Mekong Delta [7]. F is moderately toxic to fish as it inhibits the acetylcholinesterase (AChE) enzyme, which is responsible for regulating neuronal communication by breaking down the neurotransmitter acetylcholine in the synaptic cleft [9,10,11]. F disrupts the neurotransmission, leading to behavioral changes, reduced swimming ability, and respiratory problems in fish [12]. Unlike organophosphates, F binds reversibly to AChE, allowing affected fish to recover more quickly [13]. It exhibits high water solubility (420 mg/L) [14], enabling it to readily enter surface water through runoff and leach into the groundwater [15]. This mobility results in the presence of its residues across various environmental compartments and their accumulation in non-target species, posing significant risks to aquatic animal health and the overall ecosystem integrity. Toan et al. [16] and Chau et al. [17] indicated that residues of F have been frequently detected in water in rice fields, inland canals, and rivers in the Mekong Delta. Tam, et al. [18] found that one day after application, the water concentrations of F in the rice fields were 32.8 ± 19.2 µg/L. These levels significantly impacted the brain acetylcholinesterase (AChE) activity in *Anabas testudineus*, and there are reasons to believe that the F used in rice farming could have an impact on aquatic organisms, including fish, living in the water of the rice fields. The objective of this study was to investigate the acute and sublethal effects of the insecticide fenobucarb on the swimming patterns, brain AChE activity, food intake, food conversion ratio, and growth performance of silver barb (*Barbonymus gonionotus*). The aim was also to provide better insights into the recovery rate of the AChE activity and how it is influenced by the availability of food, which often can be a critical stress factor for wild fish. Silver barb was chosen as an experimental fish due to its status as a native species frequently inhabiting rice fields, where it lives, reproduces, and is continuously exposed to agrochemicals throughout its life cycle [19]. Furthermore, it is a valuable food source, recognized for its high quality, and plays a significant role in supporting the livelihoods and the wellbeing of local communities in the Mekong Delta [20,21,22].

## 2. Materials and Methods

### 2.1. Test Animals

Healthy fingerlings of silver barb, *B. gonionotus* (2–5 g/fish), were purchased at the Khoi Vy hatchery in Nga Bay City, Hau Giang Province, and transported to a wet laboratory at the College of Environment and Natural Resources, Can Tho University, Vietnam. The fish were acclimatized to the laboratory conditions in a 600 L fiberglass tank with dechlorinated tap water and continuous aeration to ensure a dissolved oxygen level of above 5 mg/L for 20 days. During the period of acclimatization, the fish were fed twice a day with commercial floating pelleted feed containing 35% protein, produced by the C.P Vietnam Livestock Joint Stock Company, Bien Hoa province, Vietnam. The fiberglass tank was thoroughly cleaned each day, both before feeding and four hours afterward. To eliminate leftover food and waste, approximately 50% of the water was replaced daily. Feeding was halted one day prior to the start of the experiment.

### 2.2. Insecticide

Commercial-grade F (50%), trade name Excel Basa 50EC (2-sec-Butylphenyl N-methylcarbamate, C_12_H_17_NO_2_), was purchased from the limited liability company Viet Thang, Long Bien district, Ha Noi, Vietnam. The commercial product was diluted with distilled water to achieve the stock solution. The accuracy of the nominal concentration was checked with gas chromatography. The stock solution was further diluted to obtain the final treatment concentrations of F.

### 2.3. Experimental Design

#### 2.3.1. AChE Sensitivity Tests

The acetylcholinesterase (AChE) sensitivity test was conducted in 60 L fiberglass tanks, each filled with 20 L of tap water that had been aerated overnight to remove chlorine residues. The experiment included a control and three F concentrations: 1% (T1), 10% (T10), and 20% (T20) of the LC_50_-96h value, corresponding to 0.13, 1.27, and 2.54 ppm of F, respectively. The LC_50_-96h value of F on silver barb (12.71 ppm) was selected, based on the research result of a previous study [23]. The treatment concentrations were prepared as previously described. The control and all the treatments were performed in three replicates with simultaneous trials. Each replicate consisted of 20 fish (2.39 ± 0.05 g/fish), randomly selected from the holding tank. Throughout the 96 h experiment, no feeding, aeration, or water exchange occurred. Dissolved oxygen (DO), pH, and temperature were recorded daily at two intervals: 6:00–7:00 a.m. and 2:00–3:00 p.m. At intervals of 3, 6, 9, 12, 24, 48, 72, and 96 h, two fish were randomly picked and removed from each experimental tank, euthanized on ice, and analyzed for AChE activity within 12 h.

#### 2.3.2. Effects of Fenobucarb on Silver Barb

An experiment was set up to determine the effect of F on the survival rate (SR), feed intake (FI), feed conversion ratio (FCR), and growth of silver barb fingerlings, using the following formulas:

Survival rate (SR): The fish were observed daily, and their mortality was recorded. The survival rate was calculated at the end of the experiment using:

$$SR\;\left(\%\right)=\frac{Total\;number\;of\;fish\;at\;final}{Total\;number\;of\;fish\;initial}\times 100$$

Feed intake (FI): The feed that was provided and left over feed were measured daily. The feed intake was calculated as:

$$FI\;(g/fish/day)=\frac{Total\;feed\;provided\;(g)\;-\;Feed\;leftover\;(g)}{Number\;of\;fish}$$

Feed conversion ratio (FCR): The FCR was determined to evaluate feed efficiency, using:

$$FCR=\frac{Total\;feed\;intake}{Total\;weight\;gain}$$

Specific growth rate (GR): the fish weight was measured at the beginning and the end of the study to calculate the specific growth rate (SGR):

$$SGR\;(\%/day)=\frac{\left(ln(Final\;weight)\;-\;ln(Initial\;weight)\right)}{Duration\;(days)}\times 100$$

The experiment was conducted in 600 L composite tanks containing 200 L of tap water, with three simultaneous replicates of three treatments corresponding to 1% (T1), 10% (T10), and 20% (T20) of the LC_50_-96h concentration. Each replicate contained 30 fish (2.39 ± 0.05 g/fish). The accuracy of the treatment concentrations was checked with gas chromatography one hour before the experimental setup and found to be 0.10 (T1), 1.25 (T10), and 2.19 (T20) (Table 1). The first day after placing the fish in the tanks, there was no feeding, aeration, or water exchange. After 24 h, the fish were fed twice a day, at 8:00 a.m. and 4:00 p.m., with commercial pelleted feed containing 30% protein at 5% of their body weight, and the water was continuously aerated. Thirty min after feeding, all the uneaten feed was taken out and weighed to calculate the FI and to avoid water pollution. Every 15 days, all the water in all the tanks was replaced with water containing the F concentration of each treatment. The experiment was carried out over 60 days. The growth rate, weight, and length were measured on day 1 and every 15 days throughout the experiment and used to calculate the FCR and the specific growth rate (SGR). The feeding was stopped 1 day before the measurement. In addition, the health and abnormalities of the experimental fish were monitored daily, and the dead fish were recorded and weighed to calculate the cumulative mortality. Throughout the experiment, the dissolved oxygen (DO), pH, and temperature were recorded daily using a pH meter (HANNA HI8314, Hanna Instruments, Binh Thanh district, Ho Chi Minh City, Vietnam) and a DO meter (HANNA HI9146, Hanna Instruments, Binh Thanh district, Ho Chi Minh City, Vietnam). Measurements were taken twice daily, once in the morning (6:00–7:00 a.m.) and once in the afternoon (2:00–3:00 p.m.).

#### 2.3.3. Recovery Test

The AChE recovery test was carried out in 60 L fiberglass tanks, each filled with 20 L of tap water that had been aerated overnight to remove chlorine residues. The experiment included three simultaneous replicates, consisting of a control group and two F concentrations: 10% (T10) and 20% (T20) of the LC_50_-96h value, corresponding to 1.27 and 2.54 ppm of F, respectively. The treatment concentrations were prepared as described earlier. Each replicate included 30 fish (2.39 ± 0.05 g/fish), which were randomly selected from the holding tank. After 48 h, all the water in the experimental tanks was replaced with clean water that was free from F. The fish in each tank were divided into two tanks (15 fish/tank). The first group, including 1T10 and 1T20, were fed daily, while the other group, including 3T10 and 3T20, were only fed every third day. The fish were provided with a commercial floating pelleted feed containing 35% protein and 1.5% lipid. Feeding was conducted twice daily, at a rate of 5% of their wet body weight. Thirty min after feeding, all the uneaten feed was taken out to avoid water pollution. Two fish were collected from each experimental tank at 48 h (prior to water exchange) and on days 1, 3, 5, 7, and 14. The fish were euthanized on ice and analyzed for AChE activity within 12 h.

#### 2.3.4. Abnormal Physiological Expression Test

The experiment was conducted in 50 L fiberglass tanks, each holding 20 L of tap water. It included three parallel replicates for each of three F concentrations, 12.2, 13.5, and 15.0 ppm, and a control group, to examine the abnormal physiological expressions and mortality of silver barb being exposed to F. Each replicate consisted of 15 fish, which were randomly selected from a fiberglass holding tank and assigned to the various concentrations. During the experiment, the abnormal behavior of the tested fish was monitored, including (1) abnormal swimming (swimming backwards and disoriented); (2) belly flip (fish sinks to the bottom of the tank, but gills are still moving); and (3) mortality (fish lies at the bottom of the tank, gills have stopped moving). The abnormal behavior was recorded with reference to the time of its appearance and the exposure concentration, and then the fish were immediately taken out and euthanized on ice. Each time, 5 fish for each symptom (15 fish/treatment and control) were collected to measure the enzyme AChE inhibition level.

### 2.4. AChE Assay

The brain was promptly excised and placed in a pre-weighed Eppendorf tube on ice to determine its weight. Following this, each brain was homogenized in 6 mL of 0.1 M phosphate buffer (pH 7.4, prepared by mixing monobasic and dibasic sodium hydrogen phosphate) while kept on ice, using a glass homogenizer. The homogenates were then transferred to 10 mL glass tubes and subjected to centrifugation at 2000× *g* for 20 min at 4 °C (Centrifuge 4k15; Sigma, Osterode am Harz, Germany). After centrifugation, 1.5 mL of the supernatant was carefully transferred to an Eppendorf tube and kept on ice, awaiting the acetylcholinesterase (AChE) activity analysis, which was performed within 12 h. The AChE activity was assessed following the procedure established by Ellman, et al. [24]. All the measurements were carried out in a temperature-controlled room, set at 25 °C. For each assay, a cuvette was prepared with 2.65 mL of 0.1 M phosphate buffer (pH 7.4) and 100 µL of 3 mM 5,5′-dithiobis (2-nitrobenzoic acid) (Sigma Aldrich Chemie, Steinheim, Germany). Just before the assay, 50 µL of 10 mM acetylthiocholine iodide (Sigma Aldrich Chemie, Steinheim, Germany) and 200 µL of the supernatant were added and mixed thoroughly. Control blanks, prepared with 200 µL of buffer instead of the supernatant, were run in duplicate for each sample. The AChE enzyme activity was determined using a UV/Vis spectrophotometer (model UV2 2000E; ATI Unicam, Cambridge, UK), which monitored the reaction over 10 cycles of 3 min and 18 s each, with an automatic interval of 22 s, at a wavelength of 412 nm, where the absorbance increase over time was linear. The activity of AChE was then calculated based on the rate of absorbance per minute.

### 2.5. Statistical Analysis

Differences in the AChE activity, survival rate, feed intake, food conversion ratio, specific growth rate, and weight between the treatments were analyzed using a oneway analysis of variance with Dunnett’s test for post-hoc comparisons after checking for normality and the homogeneity of the variance. SPSS for windows (version 17.0, SPSS Inc., Chicago, IL, USA) was used to analyze the data.

## 3. Result

### 3.1. AChE Sensitivity Test

**Figure 1:**
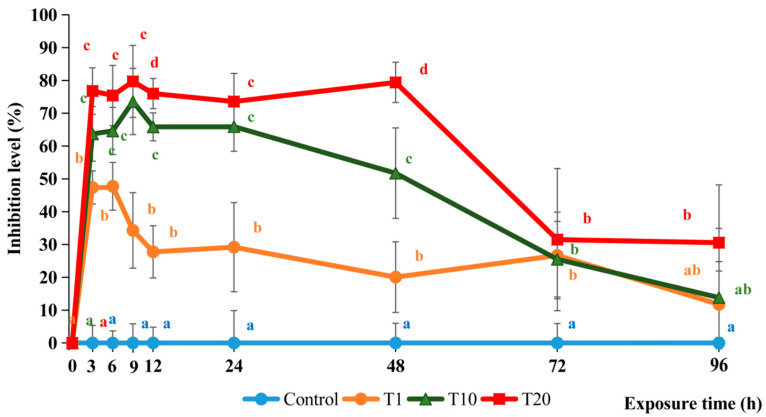
Brain acetylcholinesterase (AChE) inhibition in silver barb fingerlings exposed to 0.13 (T1), 1.27 (T10), and 2.54 (T20) ppm of F. The vertical bars show the standard deviations. Points representing samples collected at the same time with different letters are significantly different (*p* < 0.05) from each other. Each data point corresponds to the mean of six samples.

After 3 h, the AChE activity in the F treatments ranged between 4.26 and 9.65 μM/g, showing statistically significant reductions compared to the control group (*p* < 0.05). The AChE inhibition levels in T10 and T20 were 63.7 and 76.8%, respectively, and were statistically significantly higher than the 47.4% inhibition levels in T1 (*p* < 0.05) (Figure 1).

After 9 h, the AChE inhibition levels in T10 and T20 peaked at 73.6% and 79.7%, respectively, while the levels in T1 had decreased to 34.3%. After 72 h, the AChE inhibition levels in all the treatments had decreased to around 30% but were still significantly higher than the control (*p* < 0.05). After 96 h, the AChE inhibition levels in T1, T10, and T20 were 11.8%, 13.8%, and 30.6%, respectively, and only T20 was significantly higher than the control (*p* < 0.05).

### 3.2. Effect of Fenobucarb on Survival, Feed Intake, FCR, and Growth of Silver Barb

Water quality parameters, including temperature, pH, dissolved oxygen (DO), N-NH_4_^+^, and N-NO_2_^−^, were monitored throughout the experiment to evaluate the environmental conditions under the influence of F (Table 1).

The temperature was consistent across all the treatments, with values ranging from 26.0 ± 0.09 °C to 26.1 ± 0.09 °C, and there were no significant differences among the treatments (*p* > 0.05). The DO levels remained stable across the treatments, with values ranging from 6.71 ± 0.05 mg/L to 6.82 ± 0.04 mg/L. This indicates that the dissolved oxygen levels (>6.0 mg/L) in the experimental system were not affected by F and fully met the requirements for the normal growth and development of the fish. The N-NH_4_^+^, concentrations were similar across the treatments, with values ranging from 1.63 ± 0.16 mg/L to 1.77 ± 0.18 mg/L. Similarly, the N-NO_2_^−^ levels did not show significant differences between the groups, although the level of T10 was slightly higher than the other treatments (2.51 ± 0.33 mg/L). The pH in the F treatments was significantly higher than in the control (*p* < 0.05). The results showed that the levels of these parameters were within the optimal range for the healthy growth and development of silver barb [25].

Analytical results showed that the actual concentrations of F were 0.10, 1.25, and 2.19 µg/L, respectively, which were 74.8%, 98.4%, and 86.2% of the nominal concentrations, respectively (Table 2). The concentration of F in the control was below the analytical detection limit (0.0001 µg/L).

#### 3.2.1. Survival Rate (SR)

The fish mortalities in T10 and T20 occurred during the first and second replacements of the water containing the test concentration of F, and there were no more mortalities found after the third and fourth water replacements. At day 60, there were still no fish mortalities in the control or in T1, while the mortality rates in the higher-concentration treatments remained at 1.1%, with no significant differences between the treatments (*p* > 0.05) (Table 3).

#### 3.2.2. Feed Intake (FI)

**Figure 2:**
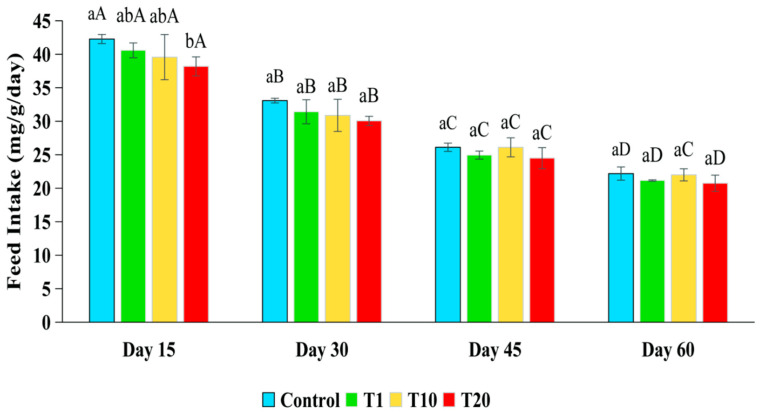
Amount of feed consumed (mg/g body weight/day) by silver barb fingerlings exposed to 0.10 (T1), 1.25 (T10), and 2.19 (T20) ppm of F. The vertical bars show the standard deviations. Bars with different superscript letters a–b show that the means were significantly different among the treatments at the same sampling time (*p* < 0.05). Bars with different superscript letters A–D show that the means were significantly different at the different sampling times of each treatment (*p* < 0.05).

During the first 15 days, the fish in the control had the highest FI (4.3 mg/g/day), which was significantly higher than the lowest FI of the fish in T20 (*p* < 0.05) but did not differ compared to the FI of the fish in T1 and T10 (*p* > 0.05) (Figure 2). The FI of the fish in all the treatments reduced gradually between days 30 and 60, but there were no statistically significant differences between the treatments and the control (*p* > 0.05). This suggested that exposure to F for 60 days did not affect the FI of silver barb.

#### 3.2.3. Food Conversion Ratio (FCR)

**Figure 3:**
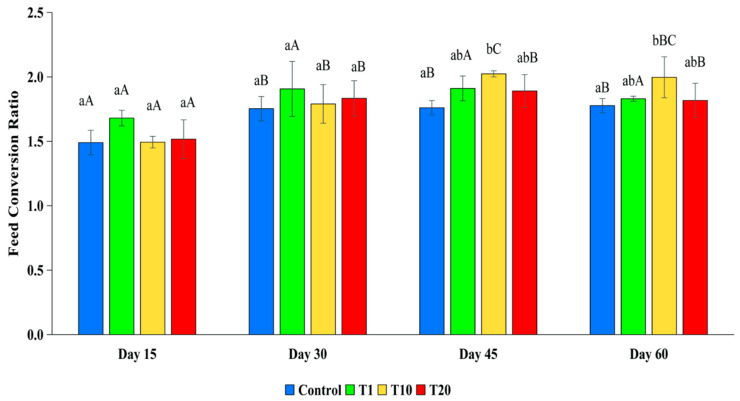
Feed conversion ratio of silver barb fingerlings exposed to 0.10 (T1), 1.25 (T10), and 2.19 (T20) ppm of F. The vertical bars show the standard deviations. Bars with different superscript letters a–b show that the means were significantly different among the treatments at the same sampling time (*p* < 0.05). Bars with different superscript letters A–C show that the means were significantly different at the different sampling times of each treatment (*p* < 0.05).

The FCR for the fish in the control was lower compared to the FCR for the fish in all the F treatments throughout the experiment, although the differences were only statistically significant between the control and T10 at day 45 and day 60 (*p* < 0.05) (Figure 3).

#### 3.2.4. Specific Growth Rate (SGR)

**Figure 4:**
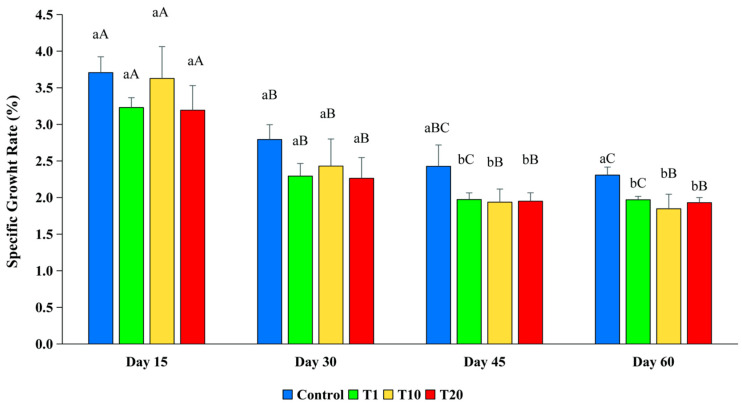
The specific growth rate of silver barb fingerlings exposed to 0.10 (T1), 1.25 (T10), and 2.19 (T20) ppm of F. The vertical bars show the standard deviation. Bars with different superscript letters a–b show that the means were significantly different among the treatments at the same sampling time (*p* < 0.05). Bars with different superscript letters A–C show that the means were significantly different at the different sampling times of each treatment (*p* < 0.05).

The SGR of the fish in the control was higher than the SGR of the fish in all the F treatments during the whole experiment, but the differences were only statistically significant between the control and all the F treatments at day 45 and day 60 (*p* < 0.05) (Figure 4). The SGR of the fish in all the treatments decreased throughout the experiment as a result of the increased fish body mass.

#### 3.2.5. Growth Rate

**Figure 5:**
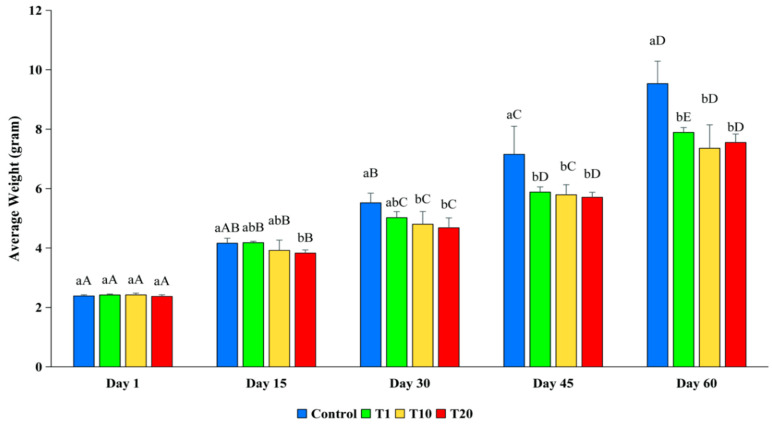
Average weight (g) of silver barb fingerlings exposed to 0.10 (T1), 1.25 (T10), and 2.19 (T20) ppm of F. The vertical bars show the standard deviations. a–b show that the means were significantly different among the treatments at the same sampling time (*p* < 0.05). A–E show that the means were significantly different at the different sampling times of each treatment (*p* < 0.05).

The initial weight (2.38–2.42 g) of the fish between the treatments was not statistically different at the start of the experiment (*p* > 0.05). However, in the later stages of the experiment, the average weight of the fish in the control was consistently higher than the weight of the fish in the F treatments (Figure 5). At day 15, the average weight of the control fish was 4.16 g, which was significantly higher than that of the fish in T20 (*p* < 0.05), but it was not significant different compared to the weight of the fish in T1 and T10, which were 4.18 g and 3.93 g, respectively (*p* > 0.05). At day 30, the weight of the fish in the control was 5.52 g, which was significantly higher than that of the fish in T10 and T20 (*p* < 0.05). At day 45 and day 60, the average weights of the fish in the control were 7.15 g and 9.53 g, respectively, which were significantly higher than the weight of the fish from all the treatments (*p* < 0.05). There were no significant differences between the treatments during these days. The result indicates that exposure to F caused a negative impact on the growth rate of silver barb fingerlings.

### 3.3. AChE Inhibition Levels That Cause Abnormal Physiological Behavior and Mortality of Silver Barb After Exposure to Fenobucarb

Before being exposed to F, all the tested fish were active and swimming normally, often in groups. After being exposed to F, the fish started to show abnormal behaviors, which were divided into three groups that often appeared in the following order:

(1) Abnormal swimming, where the fish became hyperactive, with quick, scattered swimming patterns (sometimes backward or spinning), often to the surface or into the bottom. Their gills moved fast, and convulsions occurred in some of the fish. These symptoms appeared 25 min after exposure in the treatment with 15.0 ppm of F, while they appeared after 30–55 min in the treatments with 13.5 and 12.2 ppm of F, respectively. At the onset of this symptom, the AChE activity in the fish was inhibited by 83.6%, compared to the control (*p* < 0.05) (Table 3).
(2) Belly flipping, where the fish lay still on the bottom of the tank, and the gills and fins moved only slowly. A few fish swam slowly towards the surface but then sank back to the bottom. These symptoms appeared in the treatments with 15.0 ppm and 13.5 ppm of F after 40 min and 55 min, respectively, while these symptoms appeared in the treatment with 12.2 ppm 190 min after exposure. When this symptom appeared, the AChE activity of the fish was inhibited by 84.5%, compared to the control treatment (*p* < 0.05) (Table 3).
(3) Death, where the fish’s gills were closed, and the fish no longer moved or responded to knocking on the wall of the tank. The first dead fish was found three hours after exposure in the treatment with 15 ppm of F, while the first dead fish in the treatments with 12.2 and 13.5 ppm were found 17 h 30 min and 25 h 50 min after exposure, respectively. When the fish started to die, the AChE activity in the fish was inhibited by 89.5% (Table 4).

### 3.4. AChE Recovery Test

**Figure 6:**
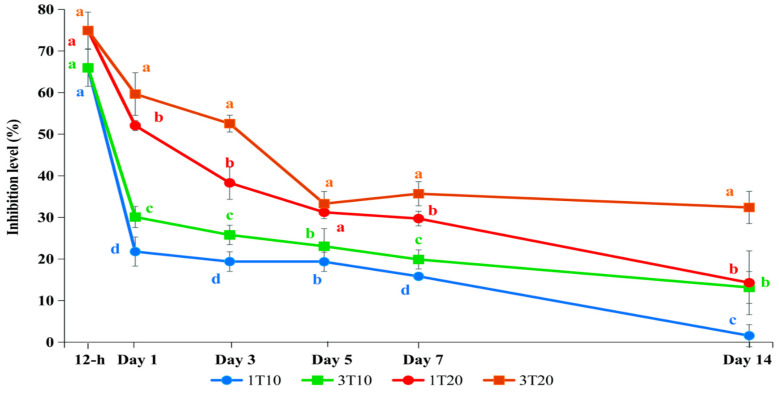
Brain acetylcholinesterase (AChE) inhibition in silver barb fingerlings exposed to 1.27 (T10) and 2.54 (T20) ppm of F and then allowed to recover in F-free water and fed with commercial feed every day (1T10 and 1T20) or every third day (3T10 and 3T20). The vertical bars show standard deviations. Points representing samples collected at the same time with different letters are significantly different (*p* < 0.05) from each other. Each data point corresponds to the mean of six samples.

Twelve hours after being exposed to F, the brain AChE inhibition levels of fish in T10 and T20 were 66.0% and 74.9% compared to the control, respectively (Figure 6). No significant differences were observed between the treatments or the feeding regimes.

The brain AChE inhibition levels in all the treatments were substantially reduced after one day in F-free water (*p* < 0.05). The reduction was influenced by both the concentration of F and the feeding frequency, and it was statistically significantly different between the treatments and the feeding regimes. The lowest inhibition level was in 1T10 (21.8%), and the highest inhibition level was in 3T20 (59.7%). The inhibition levels of the fish in both 1T10 and 3T10 were lower than 30%, a safe level for most aquatic animals, and statistically significantly lower than the other treatments. On day 14, the fish in 1T10 showed a complete recovery, while the AChE inhibition levels of the fish in 3T20 were still higher than 30%. The inhibition levels of the fish in 3T10 and 1T20 were 13.2% and 14.3% and were significantly different compared to those of 1T10 and 3T20 (*p* < 0.05). The results indicate that the AChE recovery rate in fish decreases with the decreased availability of food.

## 4. Discussion

Yen et al. [23] found that F is moderately toxic to silver barb at a LC_50_-96h value of 12.7 ppm, which is similar to the LC_50_-96h value of 11.4 ppm for climbing perch (*A. testudineus*) and snakehead fingerlings (*C. striata*) [26]. F is less toxic to silver barb than Quinalphos, at the LC_50_-96h value of 0.856 ppm [27].

Nevertheless, all the tested concentrations of F resulted in substantially reduced brain AChE activity in silver barb, indicating that F causes significant sublethal effects at F concentrations well below the LC_50_-96h value. The brain AChE activity was affected by both the concentration of F and the duration of the exposure. The brain AChE inhibition increased rapidly during the first 9 h and peaked at 73.4 and 79.7% for T10 and T20, respectively. It has been reported that levels above 70% can cause mortalities in fish due to severe neurological dysfunction and subsequent behavioral impairments [28]. However, no mortalities occurred in either T10 or T20 despite the inhibition levels in T20 remaining around 75–80% for 48 h. This could be because of the stable condition in the laboratory, which excluded other external stresses such as poor water quality and food scarcity, which can exacerbate the impact of AChE inhibition in natural environments.

The inhibition in T10 and T20 after 24 h (65.9 and 73.6%, respectively) were much higher compared to the inhibition levels in climbing perch (26% and 42%, respectively) exposed to 10% and 20% of the LC_50_-96h value of F in rice fields [29]. This could be because F disappeared quicker from the water in the field than in the laboratory [29]. Thus, the impact of F on the fish under laboratory conditions differs from the impact in the field, although the overall trend seems to be the same [30].

The inhibition level in T10 after 24 h (65.9%) was slightly higher than in climbing perch (59.5%) exposed to 10% of the LC_50_-96h value of F, but slightly lower after 72 h (25.5% and 45.7% for silver barb and climbing perch, respectively) [31]. This indicates that the impact of F on fish is quite similar between species, at least when they have similar LC_50_-96h values [26].

At the end of the experiment (96h), the AChE activity of the fish exposed to F recovered to safe inhibition levels (<30%), except for the fish in the T20 treatment, which remained at 30.6%. The decreased inhibition suggests that the fish may have increased their protein synthesis to compensate for the AChE enzymes being blocked by F [32,33]. According to Assis, Bezerra and Luiz [13], the inhibition of the AChE activity by carbamates, such as F are quick, but after the exposure has ended, the recovery of the AChE activity in the exposed organisms is also fast [18,29,31,34]. The ester bond between the active carbamate group and the hydroxyl group of AChE is relatively weak, so some AChE can be released from the bond with the carbamate group and returned to the functional AChE enzyme without undergoing synthesis [35]. Therefore, the AChE enzyme inhibited by carbamate-based pesticides has the ability to recover quickly. Similarly to our results, many studies confirm that the AChE activity in the brain of aquatic animals can be completely recovered after being inhibited by pesticides. Tam et al. [29] noted that the enzyme AChE, inhibited in the brain of *A. testudineus* by F after exposure under laboratory and field conditions, completely recovered after 14 and 3 days, respectively. The shorter recovery time in the field was probably due to a quicker disappearance of F from the rice field water than from the water in the more controlled static laboratory experiment. Another study showed that the enzyme AChE in the brain of *A. testudineus*, inhibited by a mixture of Diazinon (D) and F, was completely recovered 14 days after the fish was moved to clean water [36]. Similarly, the AChE in the brain of *Channa striata*, being inhibited by a mixture of the active ingredients Chlorpyrifos (CPF) and F, was completely recovered 14 days after the fish was moved to clean water [37]. This despite both CPF and D being organophosphates, which tend to bind more irreversibly to the AChE enzyme than carbamates, such as F.

Our results also reveal a positive relationship between the recovery time of the inhibited fish and both the concentration of F in the water and the feeding regime implemented after the exposure. More frequent feeding provides energy to support detoxification activities and helps AChE to recover faster [38]. A lower exposure to F decreases the recovery time because of the initial lower inhibition level of AChE and the better health of the fish to activate detoxification processes. This indicates the importance of different management strategies, where a balanced supply of fish feed, together with a frequent exchange of water and the restrictive use of pesticides, can help to reduce the potential impacts of pesticides on, for example, fish farming activities. It also indicates the importance of how indirect effects, like the decreased availability of food, can impact both the response and the recovery of the fish exposed to pesticides in the field.

Although only a few fish died after 15 and 30 days of exposure in the T10 and T20 treatment, most of the fish were most likely suffering from sublethal effects. Based on the manufacturer’s pesticide label, the maximum recommended application rate of Excel Basa 50EC is 2.0 L/ha. Assuming a water depth of 20 cm in the rice field and that 50% of the applied insecticide reaches the water, the resulting concentration of F in the water would be approximately 2.5 ppm. This concentration is 19.7% of the LC_50_96h value, equivalent to the concentration of T20. Tam et al. [18] found that F sprayed at the recommended dose (0.75 kg F/ha) on rice fields resulted in an F concentration in the water of 0.13 ppm after 1 h, which is equivalent to the concentration of T1. Although these are estimates of the F concentration in the rice field water, the sublethal long-term effects found in this study clearly show that the effects from the recommended dose of Excel Basa 50EC on the health and normal development of silver barb in rice fields cannot be excluded. With many farmers using higher doses, it is quite likely that the fish in the rice fields are affected negatively by the unrestricted use of pesticides, especially considering the potential cumulative effects from the several pesticides used by farmers. Many species are also indirectly affected by pesticides and stressed by other environmental factors in the field, which makes them more sensitive in the field than under laboratory conditions [30].

Alongside the reduced brain AChE activity, the fish exhibited altered behaviors across all the F concentrations, including abnormal and erratic swimming, rapid gill movements, and belly flipping. These symptoms suggest sublethal effects on the fish nervous system. These results align with observations from various studies [39,40,41], which reported alterations in feeding behavior, swimming activity, predator avoidance, and spatial orientation as a consequence of the brain AChE inhibition induced by insecticides. Brain AChE inhibition exceeding 30% has been reported to impact the normal behavior of fish, with mortality occurring when inhibition levels surpass 70% [28,42].

After the first and second replacement of the water containing the test concentration of F, the abnormal behaviors and fish death occurred, but there was no mortality found in the third and fourth applications. One possible explanation is that the prior exposure to F triggered cellular defense mechanisms in the fish, such as the activation of heat shock proteins (HSPs) or other stress inducible proteins (SPs) [43]. These chaperones play a crucial role in safeguarding organisms from damage induced by various stressors, including high temperatures, oxygen deprivation, viral infections, and chemical pollutants like pesticides [43].

Although there were no significant differences in the food intake between the treatments at any time, the control fish exhibited a significantly lower specific growth rate and higher weight in the later stages of the experiment compared to the fish exposed to the F treatments (*p* < 0.05). The explanation could be that fish use part of the energy from food for metabolism, part is excreted through feces and urine, and the rest is accumulated for growth and reproduction [44]. In the initial phase of the experiment, no significant differences in FIs were observed across the treatments, or in other words, the energy supplied from the food for the fish was similar among the treatments. However, in the later phases of the experiment, there were increasing differences in the FCR between the treatments and the control. The main reason could be that the exposed fish spent more energy to detoxify and fight stress factors, thereby reducing the energy available for growth. Similarly to this study, other studies noted that when exposed to toxins, fish tend to increase the intensity of respiration and air breathing [29]. This means that the fish that were exposed to the higher concentrations of F spent more energy to survive, and the amount of energy that could be used for growth decreased, which increased the FCR.

## 5. Conclusions

Silver barb fingerlings exhibited a high sensitivity to F, as evidenced by the pronounced effects on their brain AChE activity when exposed to sublethal concentrations of F. The inhibition of the brain AChE activity by F increased with the concentration and peaked after 9 h at 73.6% and 79.7% for the two highest concentrations. The brain AChE activity recovery was influenced by both the level of F in the water and the availability of food. After 14 days, only the fish exposed to 10% of the LC_50_-96h value and fed daily had recovered fully. There were no statistical differences in the food intake among the treatments, but the specific growth rate was lower, while the feed conversion ratio and the average weight of the control fish were higher compared to the fish exposed to F. This was probably because the fish used extra energy to fight the stress from being exposed to F. This study indicates that the commonly applied doses of F by rice farmers in the Mekong Delta can result in sublethal levels of F in water, which impact the growth of silver barb. Thus, F and the use of other highly toxic pesticides should be restricted to protect wild fish and other aquatic organisms in the Mekong Delta.

## Figures and Tables

**Table 1:** The water parameters for the experimental effects of F on the survival, feed intake, FCR, and growth of silver barb.

	Control	T1	T10	T20
Temperature (°C)	26.0 ± 0.09 ^a^	26.0 ± 0.09 ^a^	26.1 ± 0.09 ^a^	26.1 ± 0.09 ^a^
pH	6.93 ± 0.04 ^c^	7.15 ± 0.04 ^b^	7.34 ± 0.03 ^a^	7.42 ± 0.03 ^a^
DO (mg/L)	6.82 ± 0.04 ^a^	6.77 ± 0.04 ^a^	6.71 ± 0.05 ^a^	6.79 ± 0.05 ^a^
N-NH_4_^+^ (mg/L)	1.72 ± 0.17 ^a^	1.74 ± 0.19 ^a^	1.63 ± 0.16 ^a^	1.77 ± 0.18 ^a^
N-NO_2_^−^ (mg/L)	1.72 ± 0.27 ^a^	1.94 ± 0.28 ^a^	2.51 ± 0.33 ^a^	2.05 ± 0.27 ^a^

Values with different superscript letters within the same row are significantly different (*p* < 0.05) from each other.

**Table 2:** The nominal and actual concentrations of F used in the experiment investigating the effect of Excel Basa 50EC on the SR, FI, FCR, SGR, and growth rate of silver barb fingerlings.

	F (ppm)	
Treatment	Nominal Concentration	Actual Concentration
Control	Tap water	<MDL = 0.0001
T1	0.127	0.095 (0.095–0.102)
T10	1.27	1.25 (1.24–1.26)
T20	2.54	2.19 (2.17–2.20)

**Table 3:** Survival rate of fish during experiment (%).

Treatment	Day 15	Day 30	Day 45	Day 60
Control	100 ± 0.0	100 ± 0.0	100 ± 0.0	100 ± 0.0
T1	100 ± 0.0	100 ± 0.0	100 ± 0.0	100 ± 0.0
T10	100 ± 0.0	98.9 ± 1.9	98.9 ± 1.9	98.9 ± 1.9
T20	98.9 ± 1.9	98.9 ± 1.9	98.9 ± 1.9	98.9 ± 1.9

Data are expressed as mean ± SD.

**Table 4:** AChE activity (µM/g/min) and AChE inhibition rates (%) in relation to observed abnormal behavior and death in silver barb exposed to F.

	AChE (µM/g/min)	AChE Inhibition Level (%)
Normal (control)	13.5 ± 0.65 ^a^	0
Erratic swimming	2.23 ± 0.09 ^b^	83.6
Belly flipping	2.10 ± 0.10 ^b^	84.5
Death	1.42 ± 0.09 ^b^	89.5

Data are expressed as mean ± SD. Values having different superscript letters are significantly different (*p* < 0.05).

## Data Availability

The data presented in this study are available on request from the corresponding author.
